# Lessons from failure to success on malaria elimination in the Huai River Basin in China

**DOI:** 10.1136/bmj-2024-080658

**Published:** 2025-04-22

**Authors:** Xiaobo Liu, Pi Guo, Ying Liang, Chuanwei Chen, Jince Sun, Haisheng Wu, Tianyun Su, Shengjie Lai, Qiyong Liu

**Affiliations:** 1 National Key Laboratory of Intelligent Tracking and Forecasting for Infectious Diseases, National Institute for Communicable Disease Control and Prevention, Chinese Center for Disease Control and Prevention; WHO Collaborating Centre for Vector Surveillance and Management, Beijing, China; 2 Department of Vector Control, School of Public Health, Cheeloo College of Medicine, Shandong University, Jinan, China; 3 Department of Preventive Medicine, Shantou University Medical College, Shantou, China; 4 Department of Thyroid, Breast and Hernia Surgery, General Surgery, The Second Affiliated Hospital of Shantou University Medical College, Shantou, China; 5 Yongcheng Municipal Health Commission, Yongcheng, China; 6 Guoyang Center for Disease Control and Prevention, Guoyang, China; 7 School of Public Health, LKS Faculty of Medicine, The University of Hong Kong, Hong Kong SAR, China; 8 EcoZone International, Riverside, CA, USA; 9 WorldPop, School of Geography and Environmental Science, University of Southampton, Southampton, UK; 10 Institute for Life Sciences, University of Southampton, Southampton, UK

## Abstract

**Qiyong Liu and colleagues** consider the experience of basic malaria elimination, resurgence, and re-elimination in the Huai River Basin in central China, as well as lessons learnt to help to inform countries and regions in their approaches to malaria control and elimination

Malaria is still a significant public health threat. After generations of control efforts, China was certified as a malaria-free country by the World Health Organization in June 2021.^1^ Not without its challenges, China’s experience of eliminating malaria is informative for elimination strategies in other countries and regions.^2^ ^3^ ^4^

The Huai River Basin (HRB) in central China was the epicentre of two epidemics in the 1960s and 1970s, accounting for 93.1% and 91.2% of total reported cases in China, respectively.^5^ A comprehensive control strategy focused on eliminating infection sources, supplemented by integrated mosquito control, was adopted, such that by 1987 most regions in the HRB had achieved “basic malaria elimination”—with incidence rates below 1/10 000.

Unfortunately, malaria resurged in the HRB in 2003 and peaked in 2006. At that time, 62.45% of China’s total cases (60 193 cases) were in the HRB.^5^ As a result, the government’s leadership in malaria control was re-enforced through the implementation of comprehensive measures, such as mass drug administration,^5^ case management,^6^ and sustainable vector management.^7^ Consequently, the incidence of malaria in the HRB decreased significantly. No indigenous malaria has occurred in the HRB since the end of 2012 (fig 1).

**Fig 1:**
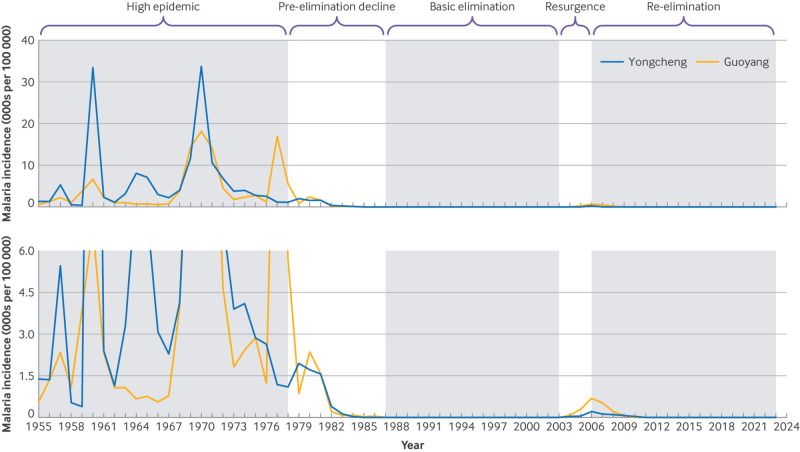
Dynamics of malaria transmission in Yongcheng City and Guoyang County, Huai River Basin, China (1955-2023). The figure illustrates the phases of high epidemic (1955-77), pre-elimination decline (1978-87), basic elimination (1988-2002), resurgence (2003-06), and re-elimination (2007-23)

Given the challenges of global malaria elimination and China’s own experience with resurgence then successful elimination, this analysis aims to draw lessons from re-emergence of malaria in the HRB, identify comprehensive measures to combat resurgence, and propose key recommendations to help other countries to prevent malaria rebounds and achieve its ultimate elimination.

## Malaria in the HRB

The HRB is located between the Yellow River and Yangtze River in central China at 30°55′-38°20′N and 111°55′-120°45′E. With a total area of 191 200 km^2^, it stretches for 1000 km from northeast to southwest, with warm temperatures in the north and a subtropical climate in the south.

The HRB’s location and climate create optimal conditions for the transmission of malaria—specifically *Plasmodium vivax,* the predominant species in this region.^4^ The prevalence of *P vivax* malaria in the HRB makes it crucial to China’s overall national strategy against malaria and useful in preventing other epidemics worldwide.

**Fig 2:**
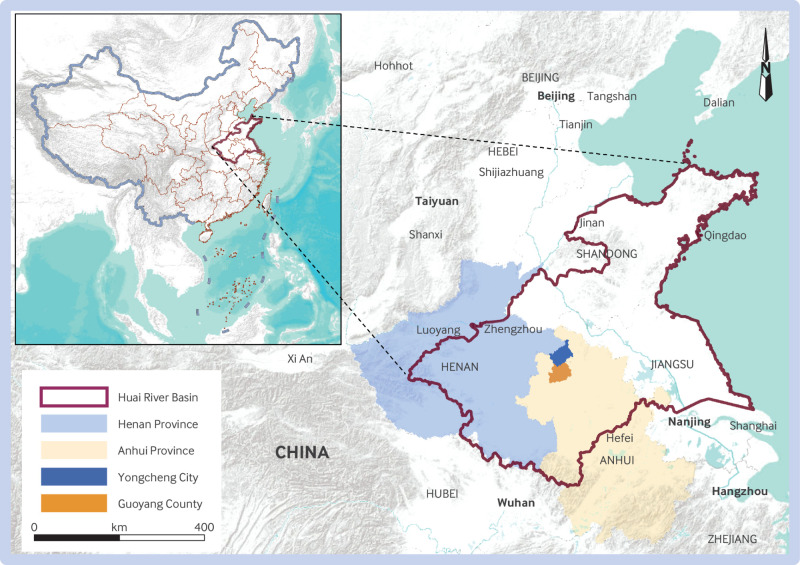
Study sites in the Huai River Basin of central China

Two sites in the HRB, Yongcheng City of Henan Province and Guoyang County of Anhui Province, are representative areas of endemic *P vivax* malaria that is transmitted by *Anopheles sinensis* Wiedmann (fig 2). Malaria incidence in Yongcheng and Guoyang achieved basic elimination in 1987, followed by a resurgence in 2003 that peaked in 2006. Given their representative nature, understanding respective journeys in malaria control, resurgence, and elimination is a useful and representative exercise.

## Contributing factors to malaria’s resurgence in the HRB

The resurgence of malaria in the HRB from 2003 was due to the convergence of multiple factors. Key factors contributing to the resurgence have been distilled as follows.

### Resource allocation away from malaria control

After the HRB achieved the basic elimination of malaria in 1987, local governments reduced funding for routine malaria control. Between 1992 and 2002, most of the local Centers for Disease Control and Prevention (CDCs) in the HRB either reduced or eliminated funding for malaria control. In Yongcheng CDC, malaria control funding was cut entirely during 1992-2002, compared with an annual average budget of ¥9820 ($2639) during 1987-91, which is equivalent to ¥46 505 ($6400; £4900; €5900) in 2024 after adjustment for annual consumer price index. In Guoyang CDC, the annual average budget of malaria control declined to ¥2736 during 1992-2002, equivalent to ¥8152 in 2024, compared with an average of ¥3080 a year during 1987-91, equivalent to ¥14 586 in 2024 (data from local CDCs, available from the corresponding author). Even though the budget during 1987-91 was already limited, the subsequent reductions had a significant effect on malaria control, particularly in the absence of proactive measures to sustain elimination. Similar trends were observed in other countries,^8^ underscoring the risks of resurgence associated with inadequate post-elimination investments in malaria control.

### Merger or closure of malaria control facilities

Public health resources are allocated on the basis of disease burdens and public health needs. In the HRB, because of the reduced funding in rural primary healthcare in connection with tight local finances, township mergers, and a low incidence of malaria for more than 10 consecutive years, some professional sectors of CDCs and public health facilities were merged or closed. This ultimately resulted in a lack of personnel to carry out malaria surveillance and alerts, with delays in the diagnosis and treatment of imported cases.^9^

### Loss of medical professionals

In Yongcheng CDC, an average of 31.8 staff members were engaged in malaria control each year from 1987 to 1991 (a total of 159 person years). From 1992 to 2002, most of these people were lost or replaced by new staff. In Guoyang CDC, an average of 6.2 staff were engaged in malaria control annually during 1987 to 1991 (31 person years in total), which dropped to 3.27 staff during 1992 to 2002 (36 person years in total) (data from local CDCs, available from the corresponding author). Massive loss of professionals seriously hampered malaria control in subsequent years.^10^ ^11^ Similarly, the reduction of village malaria workers in pre-elimination areas of Indonesia accelerated early malaria resurgence.^12^

### Low capacity for detection, diagnosis, prevention, and management of malaria cases

Malaria resurgence in the HRB underscored the consequences of complacency and the dismantling of surveillance systems after basic elimination. The low-to-no malaria cases for more than 10 years hindered both diagnostic awareness among township and village doctors and the ability to diagnose malaria among laboratory personnel. It also led to insufficient supplies of antimalarial drugs and microscopic blood examinations and equipment. For example, in 2005, the average time from onset to diagnosis of 710 malaria cases was 6.27 (standard deviation 5.58) days in Yongcheng,^5^ with the longest reaching 66 days.

### Underestimating the transmission potential of *An sinensis*

Although the incidence of malaria in the HRB was low and even basically eliminated from 1992 to 2002, the potential for resurgence caused by *An sinensis* should not have been ignored. *An sinensis* was still widely distributed, and key ecological factors suitable for malaria transmission persisted. Even in the basic elimination stage, minimal but sufficient human resources, finances, and material resources are still needed to engage in malaria surveillance and control in order to prevent resurgence.

## Comprehensive measures for a successful response after 2006

To curb the resurgence of malaria in the HRB, comprehensive control measures were implemented after 2006. The effectiveness of these measures was evidenced by a drastic decline in local epidemics since their implementation in 2007. The specific measures are as follows.

### Government empowerment and responsible leadership structure

Facing the high incidence of malaria, Yongcheng established a response leadership mechanism led by the mayor. This was a four level emergency plan in line with China’s statutory reporting of infectious diseases emergency plan, which clarified the malaria control responsibilities of public health infrastructure at all levels through signed target responsibility agreements. Similarly, Guoyang initiated a leadership mechanism headed by the deputy county magistrate in charge, established a malaria control network, and signed a letter of responsibility at all levels.

### Health administration strengthening mass drug administration

Mass drug administration is considered to be a feasible approach to reduce malaria transmission.^6^ In 2006 mass drug administration was implemented in four high incidence townships in Yongcheng, targeting 80 438 residents with chloroquine plus primaquine during spring to prevent relapses. In 2007 malaria incidence was 0.7% in the villages where all people had radical treatment and decreased by 69.6% compared with 2.3% in the previous year.^13^ During the transmission season, chemoprophylaxis with piperaquine was administered monthly to residents near water bodies. This action significantly reduced malaria incidence in Guoyang.^6^ Similar findings were obtained before the transmission season in the Gambia.^14^ In Kenya, mass drug administration significantly reduced the prevalence of* Plasmodium*,^15^ and this is regarded as a potent tool for malaria elimination.^16^ ^17^

### Hospitals of different levels carrying out blood smear examination for febrile individuals and case management

Blood smear examination for febrile people is an important way to proactively detect malaria cases. Each township health centre carried out blood smear examinations for febrile people during the malaria epidemic season in Yongcheng. Since 2005, all personnel for malaria diagnosis in township health centres have received annual training on microscopic examination techniques. Antimalarial drugs were distributed to village doctors, and then an antimalarial drug use registration system was strictly implemented to avoid misuse.

### Government organising proactive malaria vector control

Sustainable vector management was implemented in the HRB.^18^ In Yongcheng, biological control using *Bacillus sphaericus* suspension at 8 mL/m^2^ with 15 day intervals against *Anopheles* larvae was carried out in 31 administration villages that had malaria outbreaks in 2006.^19^ In total, ¥370 000 ($46 000) was used for pesticides and ¥400 000 as labour costs, equivalent to ¥557 291 and ¥602 476 in 2024, respectively. The density reduction rates of *An sinensis* larvae and adults in these villages reached 76-100% and 50-100%, respectively. In Guoyang, a risk area of 100 m around the homes of malaria cases was designated according to the dispersal range of *An sinensis*.^20^

### Health administration organising health education and training

A total of 1719 people were trained in Guoyang in 2007, including physicians, microscopists, and healthcare personnel at all levels of healthcare systems. Leveraging a rural three tier healthcare system (county and county level city health departments, township hospitals, and village clinics), residents in epidemic areas received knowledge relevant to malaria through multiple channels, including flyers, manuals, slogans, posters, Henan opera, television broadcasts, newspapers, and the annual Malaria Day event on 26 April. From 2007 to 2009, Guoyang distributed 65 000 leaflets, 156 banners, more than 5000 posters, and 1350 manuals.

### Co-funded programmes by governments and the Global Fund

Malaria control in the HRB has been jointly supported by governments and five rounds of the Global Fund to Fight AIDS, Tuberculosis and Malaria (GFFATM) since 2003. The local government invested heavily to contain the spread of malaria. For example, from 2007 to 2009, the Yongcheng municipal government allocated ¥800 000 annually, which is equivalent to ¥1 149 783 in 2024, to control malaria epidemics.

## Key recommendations for maintaining elimination and preventing reintroduction after certification

### Sustain strong political commitment, government leadership, multi-sector cooperation, and whole society participation

After elimination of malaria, maintaining political commitment, empowered leadership structures, and effective multi-sector collaboration remains critical. China has taken several concrete steps to institutionalise these practices. The National Malaria Elimination Action Plan (2010-2020) was jointly issued by 13 ministries in 2010; this was followed by the National Malaria Elimination Work Plan (2016-2020) issued by the National Health and Family Planning Commission in 2016. In 2020, 13 ministries released the Administrative Measures for the Prevention of Re-establishment of Malaria, and China’s CDC introduced the Technical Scheme for Prevention of Re-establishment of Malaria after Elimination.

A robust four level emergency response plan is now in place according to China’s statutory reporting for infectious diseases and public health emergency. Malaria reporting systems and control institutions in the HRB continue to operate efficiently, ensuring the availability of affordable services for the diagnosis and treatment of malaria.^21^ Systematic technical training has been regularly organised for healthcare providers through vocational study, continuing education programmes, and academic annual conferences, ensuring that the capacity of the malaria workforce is maintained. Public engagement is reinforced by enhancing outreach mechanisms and creating broader participation channels to promote awareness and action against malaria.^22^ ^23^ These measures have collectively ensured the re-establishment of prevention of malaria. Furthermore, other regions in China that have adopted these strategies have also successfully maintained their malaria-free status, demonstrating the efficacy and scalability of these approaches.^24^

### Ensure timely detection and management of imported cases

The risk of reintroduction remains a key concern after elimination of malaria. China has enhanced malaria surveillance in areas at risk and participates actively in regional malaria control initiatives.^21^ Continuous surveillance, rapid interventions, and follow-up for imported cases are carried out among migrating populations from high malaria prevalence regions. In-depth public education and promotion campaigns before and during the epidemic season are maintained to enhance residents’ awareness of malaria control. To mitigate the threat of reintroduction, strategies such as China’s “1-3-7 strategy”^25^ or adaptations such as Vietnam’s “2-3-7 strategy”^26^ can be implemented immediately on detection of an imported case of malaria. The 1-3-7 or 2-3-7 strategy establishes clear timelines for malaria case management: health facilities or practitioners must report a diagnosis within one or two days, health authorities are required to confirm the case and assess the transmission risk by day 3, and by day 7 appropriate measures should be taken to prevent further spread.

### Maintain a rigorous vector surveillance and response system

After elimination of malaria, continuous vector surveillance including the monitoring of insecticide resistance has been carried out continuously in the HRB. For imported cases, local CDCs do transmission risk assessments and, where necessary, immediate vector interventions are carried out by professional control agencies under the coordination of the local government. These actions are essential for preventing secondary cases and safeguarding malaria elimination. Biological control methods, such as *Bacillus sphaericus* or *Bacillus thuringiensis* subsp *israelensis* offer additional tools for effective larval source management in many countries.

### Ensuring stable funding

One of the major risk factors for malaria resurgence in a country is the reduction of funding for antimalaria programmes after elimination.^27^ Sustained, targeted funding was crucial for eliminating malaria in the HRB, China, and other regions. Following elimination, securing stable financial support is necessary to maintain core malaria control capabilities and ensure the long term stability of the malaria control workforce.^8^ To prevent the re-establishment of malaria, the Chinese government has ensured the availability of the funds for surveillance and emergency control of imported malaria cases through central transfer payment and other means.

### Strengthen international cooperation and share lessons and experience

International collaboration is key to reducing the risk of reintroduction after elimination.^28^ Lessons and experience in malaria control and elimination in the HRB and China should be summarised and shared with other countries and regions with similar situations.^29^ These experiences detail core elements of successful elimination efforts, such as leadership, proactive case detection, sustainable vector management, and workforce training.^26^ ^30^ China’s 1-3-7 strategy has been embraced by countries such as Vietnam and Cambodia, accelerating their progress towards malaria elimination.^31^ ^32^ On the basis of findings from semi-structured interviews, Malawi anticipates assistance from China in implementing mass drug administration because China possesses extensive experience in executing such programmes.^26^ Countries such as El Salvador, which experienced malaria resurgence owing to resistance of vectors to dchlorodiphenyltrichloroethane (DDT) and resistance of parasites to chloroquine,^33^ offer lessons for managing similar challenges in the future. Strengthening international cooperation and continuously sharing best practices will be crucial for informing regional and global malaria elimination efforts.

## Conclusion

After the basic elimination of malaria in the HRB, the leading reason for the resurgence of malaria in 2003 was the reduction or cessation of public health infrastructure, personnel, and budgets, resulting in low capacity for case management as well as vector surveillance and control. By examining the lessons from the containment, resurgence, and eventual elimination of malaria in China’s HRB, other countries and regions still battling malaria, especially those planning to eliminate malaria, can benefit from China’s experiences and ultimate success.

**Key messages**

Resurgence of malaria in the Huai River Basin (HRB) from 2003 to 2006 was primarily a result of the reduction and cessation of malaria and vector surveillance and control efforts after basic elimination in 1988
The successful re-elimination of malaria in the HRB was achieved through the implementation of comprehensive measures, such as strong leadership, multi-sector collaboration, mass drug administration, case management, sustainable vector management, health education and training, and stable funding
Continuously sustaining political commitment, effective case management, sustainable vector management, and stable funding are essential for maintaining malaria elimination in the HRB
China’s experience in the HRB offers valuable lessons for countries and regions that are facing the threat of endemic malaria or working to eliminate malaria or curb its resurgence

## Notes

Contributors and sources: QYL, chief expert of vector control in China CDC, conceived, designed, and supervised this analysis. XBL, PG, and YL were responsible for the preparation and analysis of data and the writing of the paper. HSW was responsible for preparation of the figures and revision of the paper. CWC and JCS were responsible for the data collection concerning the representative region in central China. TS and SJL provided constructive suggestions and comments on the draft manuscript. All authors read and approved the manuscript.

Provenance and peer review: Commissioned; externally peer reviewed.

This article is part of a collection developed in partnership with the Chinese Center for Disease Control and Prevention, Beijing. Open access fees were funded by the Gates Foundation, China. *The BMJ* commissioned, peer reviewed, edited, and made the decision to publish. Rachael Hinton and Jocalyn Clark were the lead editors for *The BMJ*.
